# Editorial: Biomedical engineering technologies and methods in antenatal medicine

**DOI:** 10.3389/fbioe.2023.1239031

**Published:** 2023-08-01

**Authors:** Giovanni Magenes, Maria G. Signorini

**Affiliations:** ^1^ Department of Electrical, Computer and Biomedical Engineering, University of Pavia, Pavia, Italy; ^2^ Centre for Health Technologies, University of Pavia, Pavia, Lombardy, Italy; ^3^ Department of Electronics, Information and Bioengineering, Polytechnic University of Milan, Milan, Lombardy, Italy

**Keywords:** fetal monitoring, fetal heart rate analysis, cardiotocography (CTG), signal processing, machine learning methods

Although pregnancy by itself should not be considered a pathological condition or a life-threatening event, the antepartum period is usually a very critical time window for both mothers and their fetuses. Pregnancy and consequently the complex interactions taking place at the level of the mother-fetus system are characterized by a peculiar trait: fast and continuous evolution in time. As a matter of fact the fetus passes from 2 cells to 3 kilos in about 9 months and the mother’s body must rapidly adapt. Therefore, careful monitoring of both subjects before delivery and during labor has been reported as an effective tool for preventing a set of possible unfavorable outcomes (Grivell et al., 2012).

In principles, a continuous tracking of pregnancy evolution in time would be the best way to proper address maternal and fetal wellbeing, however, even in the most developed countries, healthcare systems foresee an average of 3 monitoring sessions during pregnancy. Hence, these examinations are basically a series of snapshots of mother/fetus wellbeing, far apart one each other.

In this context, it is desirable to design medical devices and software tools capable of providing an effective check-up of pregnant women in several kinds of environment and daily life situations. The aim is to establish reliable health assessments in both acute (bleeding, severe nausea, abdominal pain), or non-acute situations of distress (preeclampsia, gestational diabetes, sporadic contraction, decline in fetus’ activity), for both mothers and fetuses and, eventually, to prevent them.

This Research Topic of Frontiers in Bioengineering and Biotechnology aimed at presenting new solutions for antenatal medicine from both the technological and methodological sides.

Four papers were accepted and published, covering several aspects of pre-natal monitoring.

Three of them deal with the most used fetal monitoring technology, the Cardiotocography (CTG), and try to provide new methodological tools for better identifying fetal critical conditions both in the antenatal period and during labour.

In particular, Ribeiro et al. present an exploratory work, which aims to develop intrapartum predictive models of perinatal asphyxia based on clinical parameters and fetal heart rate (fHR) indices. Perinatal asphyxia is one of the most frequent causes of neonatal mortality. Cardiotocographic signals (CTGs) in association with various clinical parameters were employed for the development of Binary logistic regression (BLR) and Naive-Bayes (NB) models. In the analyzed data (517 cases, with 15 pathological cases), he BLR model shows the best performance in predicting perinatal asphyxia.

Feng et al. propose to model intrapartum CTG from a dynamical system perspective. Empirical dynamic modeling with Gaussian processes is used, which is a Bayesian nonparametric approach for function estimation. The capacity of Gaussian processes allows for revealing causal relationships between time series. Results on real CTG recordings show that fetal heart rate (FHR) and uterine activity (UA) signals are causally related. The authors emphasize that this causal relationship and the estimated attractor manifolds can be exploited for applications in the computerized analysis of CTG records.

The contribution by Spairani et al. proposes a hybrid Machine Learning approach based on a neural architecture that receives heterogeneous data in input (quantitative parameters and images built from fHR signal) for classifying healthy and pathological fetuses. Improvements in the classification performance are achieved by setting a supervised network owing two connected branches, consisting respectively of a Multi-Layer Perceptron (MLP) and a Convolutional Neural Network (CNN). The classification system was trained with a considerable number of fHR tracings, 7,000 healthy and 7,000 pathological, recorded during prenatal ambulatory non stress tests. The classification network reaches an overall promising accuracy of 80.1%.

The fourth paper considers multi-channel fetal monitoring by means of abdominal ECG. This technology, with respect to classical US CTG fetal monitoring, can be used for long term monitoring during everyday life of the pregnant woman through wearable systems. However, fECG should be extracted from signals containing also maternal ECG, and this process implies both to select the best leads for fECG and to apply robust and reliable algorithms for its separation from maternal ECG. Baldazzi et al. propose a machine learning approach for the Signal Quality Assesment that drives channel selection to feed fully featured fECG extraction algorithms. This approach can enhance fetal QRS detection performance by identifying the most-informative channels in high-density recordings, or the best electrode positioning from repeated measurements with a low number of channels.

The research and development work presented in the papers of this special section demonstrates that advanced signal processing and wearable technologies are making big strides toward the assessment of a quantitative approach in the field of antenatal medicine. Despite a qualitative evaluation of fetal monitoring is still the most used approach in the clinical practice, we have recently witnessed growing interest and significant developments in methods focused on extracting quantitative features from fECG and/or CTG signals. We trust that the readers of Frontiers in Bioengineering and Biotechnology will find the material presented in this Research Topic to be stimulating and illustrating a good picture of the state of the art in the field.

## References

[B1] Grivell R. M. Alfirevic Z. Gyte G. M. Devane D. (2012). Antenatal cardiotocography for fetal assessment. Cochrane Database Syst. Rev. 20 (1), CD007863. 10.1002/14651858.cd007863.pub4 23235650

